# Determination of the Köthe-Toeplitz Duals over the Non-Newtonian Complex Field

**DOI:** 10.1155/2014/438924

**Published:** 2014-06-16

**Authors:** Uğur Kadak

**Affiliations:** ^1^Department of Mathematics, Faculty of Science, Gazi University, 06100 Ankara, Turkey; ^2^Department of Mathematics, Faculty of Science, Bozok University, 66100 Yozgat, Turkey

## Abstract

The important point to note is that the non-Newtonian calculus is a self-contained system independent of any other system of calculus. Therefore the reader may be surprised to learn that there is a uniform relationship between the corresponding operators of this calculus and the classical calculus. Several basic concepts based on non-Newtonian calculus are presented by Grossman (1983), Grossman and Katz (1978), and Grossman (1979). Following Grossman and Katz, in the present paper, we introduce the sets of bounded, convergent, null series and *p*-bounded variation of sequences over the complex field C* and prove that these are complete. We propose a quite concrete approach based on the notion of Köthe-Toeplitz duals with respect to the non-Newtonian calculus. Finally, we derive some inclusion relationships between Köthe space and solidness.

## 1. Introduction

It is certainly not unusual to measure deviations by ratios rather than differences. For instance, during the Renaissance, many scholars, including Galileo, discussed the following problem. Two estimates, 10 and 1000, are proposed as the value of a horse, which estimates, if any, deviates more from the true value of 100? The scholars who maintained that deviations should be measured by differences concluded that the estimate of 10 was closer to the true value. However, Galileo eventually maintained that the deviations should be measured by ratios, and he concluded that two estimates deviated equally from the true value. From the story, the question comes out this way, what if we measure by ratios? The answer is the main idea of non-Newtonian calculus which consists of many calculuses such as the classical, geometric, anageometric, and bigeometric calculus.

Bashirov et al. [1, 2] have recently concentrated on the non-Newtonian calculus and gave the results with applications corresponding to the well-known properties of derivatives and integrals in the classical calculus. Quite recently, Uzer [3] has extended the non-Newtonian calculus to the complex-valued functions and was interested in the statements of some fundamental theorems and concepts of multiplicative complex calculus and demonstrated some analogies between the multiplicative complex calculus and classical calculus by theoretical and numerical examples. In particular, Bashirov et al. [2] have studied the multiplicative differentiation for complex-valued functions and established the multiplicative Cauchy-Riemann conditions. Further, Tekin and Başar have introduced some certain sequence spaces over the non-Newtonian complex field by using ∗-calculus in [4] and many authors have introduced multiplicative calculus in biomedical image analysis and have derived non-Newtonian calculus as an alternative to the quantum calculus in [5, 6].

Following Tekin and Başar [4] we can construct the sets bs*, cs*, and *cs*_0_* consisting of the sets of all bounded, convergent, null series based on the non-Newtonian calculus, as follows:

(1) bs∗≔{x=(xk)∈ω∗:||x||bs∗=sup⁡n∈N||¨∑∗ k=0 nxk||¨<∞}, orbs∗≔{x=(xk)∈ω∗:(∑∗ k=0 nxk)∈l∞∗},cs∗≔{x=(xk)∈ω∗:(∑∗ k=0 nxk)∈c∗},cs0∗≔{x=(xk)∈ω∗:(∑∗ k=0 nxk)∈c0∗},

where ω* = {*x* = (*x*_*k*_) : *x*_*k*_ ∈ C*for all  *k* ∈ *N*}. One can conclude that the sets bs*, cs*, and *cs*_0_* are complete non-Newtonian metric spaces with the metric *d*_*∞*_^*N*^ defined by

(2) d∞N≔sup⁡n∈N{d∗(∑∗ k=0 nxk,∑∗ k=0 nyk)}.

Secondly, we introduce several sets bv*, *bv*_p_*, and *bv*_∞_* of bounded variation sequences in the sense of non-Newtonian calculus, as follows:

(3) bv∗≔{x=(xk)∈ω∗:||x||bv∗≔∑∗ k=0 ∞||¨(Δx)k′||¨<∞},dΔ(x,y)≔∑∗ k=0 ∞{d∗[(Δx)k′,(Δy)k′]}.bvp∗:={x=(xk)∈ω∗:∑∗ k=0 ∞||¨(Δx)k||¨p¨<∞},dpΔ(x,y):={∑∗ k=0 ∞d∗[(Δx)k,(Δy)k]p¨}1¨/¨p¨ (1≤p<∞),bv∞∗:={x=(xk)∈ω∗:sup⁡k∈N||¨(Δx)k||¨<∞},d∞Δ(x,y):=sup⁡k∈N{d∗[(Δx)k,(Δy)k]}.

One can easily see that the sets bv*, *bv*_p_*, and *bv*_∞_* are complete with corresponding metrics on the right-hand side with (Δ*x*)_*k*_ = *x*_*k*_ ⊖ *x*_*k*−1_, $x_{-1}=\ddot{0}$ and (Δ*x*)_*k*_′ = *x*_*k*_ ⊖ *x*_*k*+1_ for all *k* ∈ *N*.

## 2. *β*-Arithmetics and Some Related Applications

A* generator* is a one-to-one function whose domain is *R* and whose range is a subset of *B*⊆*R*, the set of real numbers. Each generator generates exactly one arithmetic, and conversely each arithmetic is generated by exactly one generator. As a generator, we choose the function *β* such that its basic algebraic operations are defined as follows:

(4) β-addx +¨ y=β{β−1(x)+β−1(y)};β-subtractionx −¨ y=β{β−1(x)−β−1(y)},β-multipl.x ×¨ y=β{β−1(x)×β−1(y)};β-division  x/¨y,xy≔β{β−1(x)÷β−1(y)},β-orderx <¨ y⟺β(x)<β(y),

for all *x*, *y* ∈ *R*(*N*), where the non-Newtonian real field *R*(*N*)≔{*β*{*x*} : *x* ∈ *R*} as in [7].

The *β*-positive real numbers, denoted by *R* ^+^(*N*), are the numbers *x* in *R* such that $\ddot{0}\;\ddot{<}\;x$; the *β*-negative real numbers, denoted by *R* ^−^(*N*), are those for which $x\;\ddot{<}\;\ddot{0}$. The *beta*-zero, $\ddot{0}$, and the *beta*-one, $\ddot{1}$, turn out to be *β*(0) and *β*(1). Further, $\beta (-1)=\ddot{-}\ddot{1}$. Thus the set of all *β*-integers turns out to be the following:

(5) Z(N)={…,β(−2),β(−1),β(0),β(1),β(2),…}={…,−¨2¨,−¨1¨,0¨,1¨,2¨,…}.

**Definition 1 .**

Let *X* be a nonempty set and let d* : *X* × *X* → *R*(*N*) be a function such that, for all *x*, *y*, *z* ∈ *X*, the following axioms hold:

 (NM1)  $d^{*}(x,y)=\ddot{0}$ if and only if *x* = *y*,
 (NM2)  d*(*x*, *y*) = d*(*y*, *x*),
 (NM3)  $d^{*}(x,y)\;\ddot{\leq }\;d^{*}(x,z)\;\ddot{+}\;d^{*}(z,y)$.

Then, the pair (*X*, d*) and d* are called a non-Newtonian metric space and a non-Newtonian metric on *X*, respectively.

**Definition 2 (see [7]).**

Let *X* = (*X*, d*) be a non-Newtonian metric space. Then the basic notions can be defined as follows.

- A sequence *x* = (*x*_*k*_) is a function from the set *N* into the set *R*(*N*). The *β*-real number *x*_*k*_ denotes the value of the function at *k* ∈ *N* and is called the *k*th term of the sequence.
- A sequence (*x*_*n*_) in a metric space *X* = (*X*, d*) is said to be ∗-convergent if for every given $\varepsilon \;\ddot{>}\;\ddot{0}$ there exist an *n*_0_ = *n*_0_(*ε*) ∈ *N* and *x* ∈ *X* such that $d^{*}(x_{n},x)\;\ddot{<}\;\varepsilon$ for all *n* > *n*_0_ and is denoted by *lim⁡_*n*→*∞*_ *x*_*n*_ = *x* or $x_{n}\overset{*}{\to }x$, as *n* → *∞*.
- A sequence (*x*_*n*_) in *X* = (*X*, d*) is said to be non-Newtonian Cauchy (∗-Cauchy) if for every $\varepsilon \;\ddot{>}\;\ddot{0}$ there is an *n*_0_ = *n*_0_(*ε*) ∈ *N* such that $d^{*}(x_{n},x_{m})\;\ddot{<}\;\varepsilon$ for all *m*, *n* > *n*_0_.

**Remark 3 .**

Let $\ddot{b}\in B\subseteq R$. Then the number $\ddot{b}\;\ddot{\times }\;\ddot{b}$ is called the *β*-square and is denoted by $b^{\ddot{2}}$. Let $\ddot{b}$ be a nonnegative number in *B*. Then $\beta \left\{\sqrt{\beta^{-1}\left(\ddot{b}\right)}\right\}$ is called the *β*-square root of $\ddot{b}$ and is denoted by $\sqrt[{..}]{\ddot{b}}$. Further for each $\ddot{b}\in B$ we can write ${\ddot{b}}^{\ddot{2}}=\beta \left\{{\left[\beta^{-1}\left(\ddot{b}\right)\right]}^{2}\right\}=\beta \{b^{2}\}$.

The *β*-absolute value of a number *x* in *B* ⊂ *R*(*N*) is defined as *β*(|*β* ^−1^(*x*)|) and is denoted by |*x*|_*β*_. For each number *x* in *B* ⊂ *R*(*N*), $\sqrt[{..}]{{\ddot{x}}^{\ddot{2}}}={\left|x\right|}_{\beta }=\beta \left(\left|\beta^{-1}\left(x\right)\right|\right)$. Then we say

(6) $$\begin{array}{c} {\left|x\right|}_{\beta }=\{\begin{array}{cc} x, & x\;\ddot{>}\;\ddot{0}\\ \ddot{0}, & x=\ddot{0}=\beta \left\{\left|\beta^{-1}\left(x\right)\right|\right\}\\ \ddot{0}\;\ddot{-}\;x, & x\;\ddot{<}\;\ddot{0}. \end{array} \end{array}$$

The non-Newtonian distance between two real numbers *x*_1_ and *x*_2_ is defined by $|x_{1}\ddot{-}x_{2}{|}_{\beta }=\beta \left\{\left|\beta^{-1}\left(x_{1}\right)-\beta^{-1}\left(x_{2}\right)\right|\right\}=\beta \left\{\left|\beta^{-1}\left(x_{2}\right)-\beta^{-1}\left(x_{1}\right)\right|\right\}={\left|x_{2}\;\ddot{-}\;x_{1}\right|}_{\beta }$. Similarly by taking into account the definition for *alpha*-generator in (6) one can conclude that the equality ${\left|x_{1}\dot{-}{\;x}_{2}\right|}_{\alpha }={\left|x_{2}\;\dot{-}\;x_{1}\right|}_{\alpha }$ holds for all *x*_1_, *x*_2_ ∈ *R*.

Now, we give a new type calculus for non-Newtonian complex terms, denoted by ∗-calculus, which is a branch of non-Newtonian calculus. From now on we will use ∗-calculus type with respect to two arbitrarily selected generator functions.

### 2.1. ∗-Arithmetics with respect to the Complex Field

Suppose that *α* and *β* are two arbitrarily selected generators and (“star-”) also are the ordered pair of arithmetics (*β*-arithmetic and *α*-arithmetic). The sets $\left(B,\ddot{+},\ddot{-},\ddot{\times },\ddot{/}\right)$ and $\left(A,\dot{+},\dot{-},\dot{\times },\dot{/}\right)$ are complete ordered fields and *beta*(*alpha*)-generator generates *beta*(*alpha*)-arithmetics, respectively. Definitions given for *β*-arithmetic are also valid for *α*-arithmetic.

The important point to note here is that *α*-arithmetic is used for arguments and *β*-arithmetic is used for values; in particular, changes in arguments and values are measured by *α*-differences and *β*-differences, respectively. The operators of this calculus type are applied only to functions with arguments in *A* and values in *B*. The ∗-limit of a function with two generators *α* and *β* is defined by

(7)  ∗lim⁡x→af(x)=b⟺∀ε >¨ 0¨, ∃δ >˙ 0˙∋|f(x)−¨ b|β ≤¨ ε,   ∀x∈A,|x −˙ a|α<˙ δ.

A function *f* is ∗-continuous at a point *a* in *A* if and only if *a* is an argument of *f* and *lim⁡_*x*→*a*_ *f*(*x*) = *f*(*a*). When *α* and *β* are the identity function *I*, the concepts of ∗-limit and ∗-continuity are identical with those of classical limit and classical continuity.

The isomorphism from *α*-arithmetic to *β*-arithmetic is the unique function *ι* (iota) that possesses the following three properties:

(i) *ι* is one to one.
(ii) *ι* is from *A* onto *B*.
(iii) For any numbers, *u* and *v* in *A*,
  (8) ι(u +˙ v)=ι(u) +¨ ι(v),  ι(u −˙ v)=ι(u) −¨ ι(v)ι(u ×˙ v)=ι(u) ×¨ ι(v),  ι(u/˙v)=ι(u)/¨ι(v);v≠0˙, u ≤˙ v⟺ι(u) ≤¨ ι(v).

It turns out that *ι*(*x*) = *β*{*α* ^−1^(*x*)} for every *x* in *A* and that $\iota (\dot{n})=\ddot{n}$ for every integer *n*. Since, for example, $u\;\dot{+}\;v=\iota^{-1}\{\iota (u)\;\ddot{+}\;\iota (v)\}$, it should be clear that any statement in *α*-arithmetic can readily be transformed into a statement in *β*-arithmetic.

Let $\alpha (a)=\dot{a}\in (A,\dot{+},\dot{-},\dot{\times },\dot{/})$ and $\beta (b)=\ddot{b}\in (B,\ddot{+},\ddot{-},\ddot{\times },\ddot{/})$ be arbitrarily chosen elements from corresponding arithmetics. Then the ordered pair $\left(\dot{a},\ddot{b}\right)$ is called a ∗-point. The set of all ∗-points is called the set of ∗-complex numbers and is denoted by C*; that is,

(9) $$\begin{array}{c} C^{*}:=\left\{z^{*}=\left(\dot{a},\ddot{b}\right)\;|\;\dot{a}\in A,\;\ddot{b}\in B\subseteq R\right\}. \end{array}$$

Define the binary operations addition (⊕) and multiplication (⊙) of ∗-complex numbers $z_{1}^{*}=({\dot{a}}_{1},{\ddot{b}}_{1})$ and $z_{2}^{*}=({\dot{a}}_{2},{\ddot{b}}_{2})$:

(10) ⊕:C∗×C∗⟶C∗(z1∗,z2∗)⟼z1∗⊕z2∗=(α{a1+a2},β{b1+b2})=(a˙1+˙ a˙2,b¨1+¨ b¨2)⊙:C∗×C∗⟶C∗(z1∗,z2∗)⟼z1∗⊙z2∗=(α{a1a2−b1b2},β{a1b2+b1a2}),

where ${\dot{a}}_{1},{\dot{a}}_{2}\in A$ and ${\ddot{b}}_{1},{\ddot{b}}_{2}\in B$.

**Theorem 4 (see [4]).**

(C*, ⊕, ⊙) is a field.

Following Grossman and Katz [8] we can give the definition of ∗-distance and some applications with respect to the ∗-calculus which is a kind of calculi of non-Newtonian calculus.

The ∗-distance d* between two arbitrary elements $z_{1}^{*}=({\dot{a}}_{1},{\ddot{b}}_{1})$ and $z_{2}^{*}=({\dot{a}}_{2},{\ddot{b}}_{2})$ of the set C* is defined by

(11) d∗:C∗×C∗⟶[¨0¨,∞)¨=B′⊂B(z1∗,z2∗)⟼d∗(z1∗,z2∗)=[ι(a˙1 −˙ a˙2)]2¨+¨(b¨1−¨ b¨2)2¨..=β{(a1−a2)2+(b1−b2)2}.

Up to now, we know that C* is a field and the distance between two points in C* is computed by the function d*, defined by (11).

**Definition 5 .**

Given a sequence (*z*_k_*) of ∗-complex numbers, the formal notation,

(12) ∑∗ k=0 ∞zk∗=z0∗⊕z1∗⊕z2∗⊕⋯⊕zk∗⊕⋯ ∀k∈N,

is called an infinite series with ∗-complex terms or simply complex *N*-series. Also, for integers, *n* ∈ *N*, the finite ∗-sums *s*_n_* = _∗_∑∑_*k*=0_^*n*^ *z*_k_* are called the partial sums of complex *N*-series. If the sequence ∗-converges to a complex number s*, then we say that the series ∗-converges and write s* = _∗_∑∑_*k*=0_^*n*^ *z*_n_*. The number s* is then called the ∗-sum of this series. If (*s*_*n*_)  ∗-diverges, we say that the series ∗-diverges or it is ∗-divergent.

**Proposition 6 (see [4]).**

For any *z*_1_*, *z*_2_* ∈ C*, the following statements hold:

(i) $\ddot{||}z_{1}^{*}⊕z_{2}^{*}\ddot{||}\;\ddot{\leq }\;\ddot{||}z_{1}^{*}\ddot{||}\;\;\ddot{+}\;\ddot{||}z_{2}^{*}\ddot{||}$ (∗*-triangle inequality).*
(ii) $\ddot{||}z_{1}^{*}⊙z_{2}^{*}\ddot{||}=\ddot{||}z_{1}^{*}\ddot{||}\;\ddot{\times }\;\ddot{||}z_{2}^{*}\ddot{||}$.
(iii) *Let* $p\;\ddot{>}\;\ddot{1}$ *and z*_k_*, *t*_k_* ∈ C* for k ∈ {0,1, 2,3,…, *n*}*. Then, *
  (13) ( ∗∑k=0n||¨zk∗⊕tk∗||¨p)1/¨p≤¨  ( ∗∑k=0n||¨zk∗||¨p)1/¨p+¨  ( ∗∑k=0n||¨tk∗||¨p)1/¨p               (Minkowski's inequality).

Following Tekin and Başar [4], we can give the ∗-norm and next derive some required inequalities in the sense of non-Newtonian complex calculus.

Let z* ∈ C* be an arbitrary element. The distance function d*(z*, θ*) is called ∗-norm of z* and is denoted by $\ddot{||}\cdot \ddot{||}$. In other words,

(14) $$\begin{array}{c} \ddot{||}z^{*}\ddot{||}=d^{*}\left(z^{*},\theta^{*}\right)=\sqrt[{..}]{{\left[\iota \left(\dot{a}\;\dot{-}\;\dot{0}\right)\right]}^{\ddot{2}}\ddot{+}{\left(\ddot{b}\;\ddot{-}\;\ddot{0}\right)}^{\ddot{2}}}=\beta \left\{\sqrt{a^{2}+b^{2}}\right\}, \end{array}$$

where $z^{*}=(\dot{a},\ddot{b})$ and $\theta^{*}=(\dot{0},\ddot{0})$. Moreover, since for all *z*_1_*, *z*_2_* ∈ C* we have $d^{*}\left(z_{1}^{*},z_{2}^{*}\right)=\ddot{||}z_{1}^{*}⊖z_{2}^{*}\ddot{||}$, d* is the induced metric from $\ddot{||}\cdot \ddot{||}$ norm.

**Definition 7 (complex conjugate).**

Let $z^{*}=(\dot{a},\ddot{b})\in C^{*}$. We define the ∗-complex conjugate ${\bar{z}}^{*}$ of z* by ${\bar{z}}^{*}=(\alpha \{a\},\beta \{-\beta^{-1}(\ddot{b})\})=(\dot{a},\ddot{-}\;\ddot{b})$. Conjugation changes the sign of the imaginary part of z* but leaves the real part the same. Thus

(15) Re(z¯∗)=Re(z∗)=(z∗⊕z¯∗)/˙2˙=a˙,Im(z¯∗)=−¨Im(z∗)=(z∗⊖z¯∗)/¨2¨=b¨.

**Remark 8 .**

(i) Let $z^{*}=(\dot{a},\ddot{b})$, $w^{*}=(\dot{c},\ddot{d})\in C^{*}$. We can give the ∗-division as

(16) z⊘w=(a˙,b¨)⊘(c˙,d¨)=(α{ac+bdc2+d2},β{bc−adc2+d2}).

(ii) Let *α* and *β* be the same generator functions and z* ∈ C*. Then the following condition holds

(17) z∗⊙z∗¯=(a˙,b¨)⊙(a˙,−¨ b¨)=(α{a2+b2},β(0))=β{a2+b2}=β{(β−1βa2+b2)2}=(||¨z∗||¨)2¨.

**Theorem 9 (see [4]).**

(C*, d*) is a complete metric space, where d* is defined by (11).

**Corollary 10 (see [4]).**

C* is a Banach space with the ∗-norm $\ddot{||}\cdot \ddot{||}$ defined by $\ddot{||}z^{*}\ddot{||}=\sqrt[{..}]{\iota {\left(\dot{a}\right)}^{\ddot{2}}\ddot{+}{\left(\ddot{b}\right)}^{\ddot{2}}}$; $z^{*}=\left(\dot{a},\ddot{b}\right)\in C^{*}$.

## 3. Completeness of the Sets of Bounded, Convergent, and Null Series over the Geometric Complex Field

Quite recently Tekin and Başar [4] have introduced the sets *l*_∞_*, c*, *c*_0_*, and *l*_p_* of all bounded, convergent, null, and absolutely *p*-summable sequences over the complex field C* which correspond to the sets *l*_*∞*_, *c*, *c*_0_, and *l*_*p*_ over the complex field *C*, respectively. That is to say,

(18) l∞∗={z∗=(zk∗)∈ω∗:sup⁡k∈N||¨zk∗||¨<∞},c∗={z∗=(zk∗)∈ω∗:∃l∈C∗∋lim⁡∗    k→∞ zk∗=l},c0∗={z∗=(zk∗)∈ω∗:lim⁡∗    k→∞ zk∗=θ∗},lp∗={z∗=(zk∗)∈ω∗:∑∗ k=1 ∞||¨zk∗||¨p¨<∞}, (1≤p<∞).

It is not hard to show that the sets *l*_∞_*, c*, *c*_0_*, and *l*_p_* are the subspaces of the space ω*. This means that *l*_∞_*, c*, *c*_0_*, and *l*_p_* are classical sequence spaces over the field C* and complete metric spaces with corresponding metrics.

**Theorem 11 (see [4]).**

The following statements hold.

(a) The sets *l*_∞_*, c*, *c*_0_*, and *l*_p_*, *p* ≥ 1, are sequence spaces.
(b) Let λ* denote any of the spaces *l*_∞_*, c*, and *c*_0_* and *z* = (*z*_k_*), *t* = (*t*_k_*) ∈ λ*. Define *d*_∞_* on the space λ* by $d_{\infty }^{*}(z,t)=\sup_{k\in N}\ddot{||}z_{k}^{*}⊖t_{k}^{*}\ddot{||}$. Then, (λ*, *d*_∞_*) is a complete metric space.
(c) The spaces *l*_∞_*, c*, and *c*_0_* are Banach spaces with the norm ||*z*||_∞_* defined by
  (19) ||z||∞∗≔sup⁡k∈N||¨zk∗||¨; z=(zk∗)∈λ∗,          λ∈{l∞,c,c0}.
(d) The space *l*_p_* is Banach spaces with the norm ||*z*||_p_* defined by
  (20) ||z||p∗≔(∑∗ k=0 ∞||¨zk∗||¨p¨)1¨/¨p¨; z=(zk∗)∈lp∗.

In the present section, we introduce the sets bs*, cs*, *cs*_0_* and bv*, *bv*_p_*, *bv*_∞_* consisting of all bounded, convergent, null series and the sets of bounded variation sequences in the sense of non-Newtonian calculus which correspond to the sets *bs*, *cs*, *cs*_0_ and *bv*, *bv*_*p*_, *bv*_*∞*_ over the complex field *C*, respectively.

**Theorem 12 .**

Let μ* denote any of the spaces bs*, cs*, and *cs*_0_* and *z* = (*z*_k_*), *t* = (*t*_k_*) ∈ μ*. Define *d*_*∞*_^*N*^ on the space μ* by

(21) d∞N:μ∗×μ∗⟶R(N)(z,t)⟶d∞N(z,t)≔sup⁡n∈N{d∗(∑∗ k=0 nzk∗,∑tk∗∗k=0   n    )}

for arbitrarily chosen *α*, *β* operators and corresponding function *ι* = *βα* ^−1^. Then, (μ*, *d*_*∞*_^*N*^) is a complete metric space.

**Proof**

Since the proof is similar to the spaces cs* and *cs*_0_*, we prove the theorem only for the space bs*. Let the ∗-sums _∗_∑∑_*k*=0_^*n*^ *z*_k_*, _∗_∑∑_*k*=0_^*n*^ *t*_k_* ∈ C*, where *z* = (*z*_k_*), *t* = (*t*_k_*) ∈ C*. Then the following metric axioms in Definition 1 are valid.

 (NM1) From (11) it can be easily obtained that
  (22) d∞N(z,t)=0¨⟺sup⁡n∈N||¨∑∗ k=0 nzk∗⊖∑tk∗∗k=0   n   ||¨=0¨⟺∑∗ k=0 nzk∗=∑∗ k=0 ntk∗⟺z=t.
 (NM2) It is trivial that the condition *d*_*∞*_^*N*^(*z*, *t*) = *d*_*∞*_^*N*^(*t*, *z*) holds.
 (NM3) We show that ∗triangle inequality in Definition 1 holds for *z* = (*z*_k_*), *t* = (*t*_k_*), *w* = (*w*_k_*) ∈ C*. In fact by taking into account Proposition 6 (i)
  (23) d∞N(z,t) =sup⁡n∈N||¨∑∗ k=0 nzk∗⊖∑∗ k=0 nwk∗⊕∑∗ k=0 nwk∗⊖∑∗ k=0 ntk∗||¨ ≤¨sup⁡n∈N||¨∑∗ k=1 nzk∗⊖∑∗ k=0 nwk∗||¨+¨ sup⁡n∈N||¨∑∗ k=0 nwk∗⊖∑∗ k=0 ntk∗||¨,d∞N(z,t) ≤¨ d∞N(z,w) +¨ d∞N(w,t).

Since the axioms (NM1)–(NM3) are satisfied, (bs*, *d*_*∞*_^*N*^) is a non-Newtonian metric space. It remains to prove the completeness of the space bs*.

Let (*x* ^*m*^) be a ∗-Cauchy sequence in bs*, where *x* ^*m*^ = {*x*_1_^(*m*)^, *x*_2_^(*m*)^,…}. Then, for every $\varepsilon \;\ddot{>}\;\ddot{0}$, there is an element *n*_0_ such that, for all *m*, *r* > *n*_0_,

(24) d∞N(xm,xr)=sup⁡n∈N{d∗(∑∗ k=0 nxk(m),∑∗ k=0 nxk(r))} <¨ ε.

A fortiori, for every fixed *k* ∈ *N* and for all *m*, *r* > *n*_0_

(25) d∗(∑∗ k=0 nxk(m),∑∗ k=0 nxk(r)) <¨ ε.

Hence, for every fixed *k* ∈ *N*, the sequence (*x*_*m*_) = {*x*_*k*_^(1)^, *x*_*k*_^(2)^,…, *x*_*k*_^(*m*)^,…} is a ∗-Cauchy sequence. Before that, by using the completeness of C* in Theorem 9, it ∗-converges; that is, $x_{k}^{\left(m\right)}\overset{*}{\to }x_{k}$ as *m* → *∞*. Using these infinitely many limits *x*_1_, *x*_2_,…, we define *x* = (*x*_1_, *x*_2_,…) and show that *x* ∈ bs*. From (25) letting *r* → *∞* and *m* > *n*_0_ we have

(26) d∗(∑∗ k=0 nxk(m),∑∗ k=0 nxk) ≤¨ ε.

Since (*x* ^*m*^) ∈ bs*, there exists $\ddot{p}\in R(N)$ such that $\ddot{||}x_{k}^{\left(m\right)}\ddot{||}\;\ddot{\leq }\;\ddot{p}$ for all *k* ∈ *N*. Thus, (26) gives together with the ∗-triangle inequality for *m* > *n*_0_

(27) d∗(∑∗ k=0 nxk,0¨) ≤¨ d∗(∑∗ k=0 nxk,∑∗ k=0 nxk(m)) +¨ d∗(∑∗ k=0 nxk(m),0¨)≤¨ ε +¨ p¨.

It is clear that (26) holds for every *k* ∈ *N* whose right-hand side does not involve *k*. Hence (*x*_*k*_) is a bounded sequence of geometric complex numbers; that is, *x* = (*x*_*k*_) ∈ bs*. Also from (26) we obtain for *m* > *n*_0_

(28) d∞N(xm,x)=sup⁡n∈N{d∗(∑∗ k=0 nxk(m),∑∗ k=0 nxk)} ≤¨ ε.

Hence, as we have seen above that $x^{m}\overset{*}{\to }x$, as *m* → *∞* for an arbitrary ∗-Cauchy sequence (*x* ^*m*^). Hence bs* is complete.

Thus it is known by Theorem 12 that the spaces bs*, cs*, and *cs*_0_* are complete metric spaces with the metric *d*_*∞*_^*N*^ induced by the norm ||·||_bs_* or ||·||_cs_*. Now, as a consequence of Theorem 12, the following corollary presents for these spaces to be Banach space.

**Corollary 13 .**

The spaces bs*, cs*, and *cs*_0_* are Banach spaces with the norm ||*x*||_bs_* defined by

(29) ||x||bs∗=||x||cs∗=sup⁡n∈N||¨∑∗ k=0 nxk||¨; x=(xk)∈λ∗,                  λ∈{bs,cs,cs0}.

To avoid undue repetition in the statements we give the next theorem which is on the complete metric space bv* without proof since the proof can be obtained similarly as Theorem 12.

**Theorem 14 .**

Let *d*_Δ_ be defined on the space bv* by

(30) dΔ:bv∗×bv∗⟶R(N)(x,y)⟶dΔ(x,y)≔∑∗ k=0 ∞{d∗[(Δx)k′,(Δy)k′]},

where *x* = (*x*_*k*_), *y* = (*y*_*k*_) ∈ bv*, and (Δ*x*)_*k*_′ = *x*_*k*_ ⊖ *x*_*k*+1_. Then, (bv*, *d*_Δ_) is a complete metric space. Similarly, one can conclude that the other bounded variation sets (*bv*_p_*, *d*_*p*_^Δ^) and (*bv*_∞_*, *d*_*∞*_^Δ^) are also complete.

**Corollary 15 .**

The space bv* is a Banach space with the norm ||*x*||_bv_* defined by

(31) ||x||bv∗≔∑∗ k=0 ∞||¨(Δx)k′||¨, (Δx)k′=(xk⊖xk+1),∀k∈N.

## 4. The Duals of the Sets of Sequences over the Geometric Complex Field

The idea of dual sequence space which plays an important role in the representation of linear functionals and the characterization of matrix transformations between sequence spaces was introduced by Köthe and Toeplitz [9], whose main results concerned alpha-duals. An account of the duals of sequence spaces can be found in Köthe [10]. One can also know about different types of duals of sequence spaces in Maddox [11].

In this section, we focus on the alpha-, beta-, and gamma-duals of the classical sequence spaces over non-Newtonian complex field. For λ*, μ* ∈ C*, the set *S*(λ*, μ*), defined by

(32) S(λ∗,μ∗)≔{w=(wk)∈ω∗:w⊙z=(wk⊙zk)∈μ∗,∀z=(zk)∈λ∗},

is called the multiplier space of λ* and μ* for all *k* ∈ *N*. One can easily observe for a sequence space ν* of ∗-complex numbers that the inclusions *S*(λ*, μ*) ⊂ *S*(ν*, μ*) if ν* ⊂ λ* and *S*(λ*, μ*) ⊂ *S*(λ*, ν*) if μ* ⊂ ν* hold.

Firstly, we define the alpha-, beta-, and gamma-duals of a set λ* ⊂ ω* which are, respectively, denoted by {λ*}^*α*^, {λ*}^*β*^, and {λ*}^*γ*^, as follows:

(33) {λ∗}α:={w=(wk)∈ω∗:w⊙z=(wk⊙zk)∈l1∗,∀z=(zk)∈λ∗},{λ∗}β:={w=(wk)∈ω∗:w⊙z=(wk⊙zk)∈cs∗,∀z=(zk)∈λ∗},{λ∗}γ:={w=(wk)∈ω∗:w⊙z=(wk⊙zk)∈bs∗,∀z=(zk)∈λ∗},

where (*w*_*k*_⊙*z*_*k*_) is the coordinatewise product of ∗-complex numbers *w* and *z* for all *k* ∈ *N*. Then {λ*}^*β*^ is called beta-dual of λ* or the set of all convergence factor sequences of λ* in cs*. Firstly, we give a remark concerning the ∗-convergence factor sequences.

Throughout the text, we also use the notation “<” for a non-Newtonian linear subspace which was created in [7].

**Remark 16 .**

Let *∅* ≠ λ* ⊂ ω*. Then the following statements are valid.

- {λ*}^*β*^ is a sequence space if φ* < {λ*}^*β*^ < ω*, where $\varphi^{*}:=\{x=(x_{k}):\exists N\in N,\forall k\geq N,x_{k}=\ddot{0}\}$.
- If λ* ⊂ μ* ⊂ ω*, then {μ*}^*β*^ < {λ*}^*β*^.
- λ* ⊂ {λ*}^*ββ*^ : = ({λ*}^*β*^)^*β*^.
- {φ*}^*β*^ = ω* and {ω*}^*β*^ = φ*.

**Proof**

Since the proof is trivial for the conditions (b) and (c), we prove only (a) and (d). Let *w* = (*w*_*k*_), *m* = (*m*_*k*_), and *n* = (*n*_*k*_)∈{λ*}^*β*^.

(a) It is trivial that {λ*}^*β*^ < ω* holds from the hypothesis. We show that *m* ⊕ *n* ∈ {λ*}^*β*^ for *m*, *n* ∈ {λ*}^*β*^. Suppose that *l* ∈ λ*. Then (*m*_*k*_⊙*l*_*k*_) ∈ cs* and (*n*_*k*_⊙*l*_*k*_) ∈ cs* for all *l* ∈ λ*. We can deduce that
  (34) $$\begin{array}{c} \left(\left(m_{k}⊕n_{k}\right)⊙l_{k}\right)=\left(m_{k}⊙l_{k}\right)⊕\left(n_{k}⊙l_{k}\right)\in cs^{*},\;\forall l\in \lambda^{*}. \end{array}$$
 Hence, *m* ⊕ *n* ∈ {λ*}^*β*^. Now, we show that *t*⊙*w* ∈ {λ*}^*β*^ for any *t* ∈ C* and *w* = (*w*_*k*_)∈{λ*}^*β*^, since (*w*_*k*_⊙*l*_*k*_) ∈ cs* for all *l* ∈ λ*. Combining this with ((*t*_*k*_⊙*w*_*k*_)⊙*l*_*k*_) = *t*_*k*_⊙(*w*_*k*_⊙*l*_*k*_) ∈ cs* for all *l* ∈ λ* we get *t*⊙*w* ∈ {λ*}^*β*^. Therefore, we have proved that {λ*}^*β*^ is a subspace of the space ω*.
(d) Using (a) we need only to show {ω*}^*β*^ ⊂ φ*. Suppose that *w* = (*w*_*n*_)∈{ω*}^*β*^ and *z* = (*z*_*n*_) are given with ∗-division by *z*_*n*_⊙*w*_*n*_ = 1* if *w*_*n*_ ≠ 0* and *z*_*n*_ : = 1* otherwise. By taking into account the set φ* from inclusion (a), then there exists an integer *N* ∈ *N* for all *n* ≥ *N* such that $w_{n}=\ddot{0}$. Thus, we have
  (35) ∑∗     n=1     ∞wn⊙zn=[(w1⊙z1)⊕⋯⊕(w1⊙z1)] ⊕(wN⊙zN)⊕(wN+1⊙zN+1) ⊕θ∗⊕θ∗⊕⋯=∑∗     n=1    ∞1∗<∞.
 Further, *w*⊙*z* ∈ cs* implies that *w* ∈ φ*. The rest is an immediate consequence of this part and we omitted the details.

**Theorem 17 .**

The following statements hold:

- {*c*_0_*}^*β*^ = {c*}^*β*^ = {*l*_∞_*}^*β*^ = *l*_1_*.
- {*l*_1_*}^*β*^ = *l*_∞_*.

**Proof**

(a) Obviously {*l*_∞_*}^*β*^ ⊂ {c*}^*β*^ ⊂ {*c*_0_*}^*β*^ by Remark 16 (b). Then we must show that *l*_1_* ⊂ {*l*_∞_*}^*β*^ and {*c*_0_*}^*β*^ ⊂ *l*_1_*. Now, consider that *w* = (*w*_*k*_) ∈ *l*_1_* and *z* = (*z*_*k*_) ∈ *l*_∞_* are given. By taking into account the cases (c)-(d) of Theorem 11, we have

(36) ∑∗    k=0      n||¨wk⊙zk||¨ ≤¨ ||z||∞∗ ×¨ ||w||1∗<∞, ∀k∈N,                  (ζ∈{α,β,γ})

which implies that *w*⊙*z* ∈ cs*. So the condition *l*_1_* ⊂ {*l*_∞_*}^*β*^ holds.

Conversely, for a given *y* = (*y*_*k*_) ∈ ω*∖*l*_1_*, we prove the existence of an *x* ∈ *c*_0_* with *y*⊙*x* ∉ cs*. According to *y* ∉ *l*_1_* we may confirm an index sequence (*n*_*p*_) which is strictly increasing with $n_{0}=\ddot{0}$ and ∑∗∑k=np-1np-1||¨yk||¨ >¨ p;(p∈N). By taking into account Remark 8 (i), if we define *x* = (*x*_*k*_) ∈ *c*_0_* by *x*_*k*_ : = (sgn*y_*k*_⊘*p*), the non-Newtonian complex signum function is defined by

(37) $$\begin{array}{c} \text{sg}{\text{n}}^{*}\left(y\right)≔\{\begin{array}{cc} \bar{y}⊘\ddot{||}y\ddot{||}, & y\neq \ddot{0},\\ \ddot{0}, & y=\ddot{0}, \end{array} \end{array}$$

where $\bar{y}$ is given complex conjugate in Definition 7 for all *y* = (*y*_*k*_) ∈ C*. Finally, by using Remark 8 (ii) taking the generators *α* = *β*, we get

(38) ∑∗ k=np−1 np−1yk⊙xk=∑∗k=np−1np−1[yk⊙(sgn∗yk⊘p)]=1p ×¨∑∗ k=np−1 np−1||¨yk||¨ ≥¨ 1¨

for all *n*_*p*−1_ ≤ *k* < *n*_*p*_. Therefore *y*⊙*x* ∉ cs* and thus *y* ∉ {*c*_0_*}^*β*^. Hence {*c*_0_*}^*β*^ ⊂ *l*_1_*.

(b) From the condition (c) of Remark 16 we have *l*_∞_* ⊂ ({*l*_∞_*}^*β*^)^*β*^ = {*l*_1_*}^*β*^ since {*l*_∞_*}^*β*^ = *l*_1_*. Now we assume the existence of a *w* = (*w*_*n*_)∈{*l*_1_*}^*β*^∖*l*_∞_*. Since *w* is unbounded there exists a subsequence (*w*_*n*_*k*__) of (*w*_*n*_) and we can find a real number (*k* + 1)^2^ such that $\ddot{||}w_{n_{k}}\ddot{||}\;\ddot{\geq }\;{\left(k+1\right)}^{2}$ for all *k* ∈ *N*_1_. The sequence (*x*_*n*_) is defined by *x*_*n*_ : = (sgn*(*w*_*n*_*k*__)⊘(*k* + 1)^2^) if *n* = *n*_*k*_ and $\ddot{0}$ otherwise. Then *x* ∈ *l*_1_*. However,

(39) ∑∗ nwn⊙xn=∑∗ k1(k+1)2⊙||¨wnk||¨ ≥¨∑∗ k1=∞.

Hence, *w* ∉ {*l*_1_*}^*β*^, which contradicts our assumption and {*l*_1_*}^*β*^ ⊂ *l*_∞_*. This step completes the proof.

In addition to the statements in Remark 16 we make the following remarks which are immediate consequences of the definition of the *ζ*-duals (*ζ* ∈ {*α*, *β*, *γ*}).

**Remark 18 .**

Let *∅* ≠ λ* ⊂ ω*. Then the following statements are valid:

- φ* < {λ*}^*α*^ < {λ*}^*β*^ < {λ*}^*γ*^ < ω*; in particular, {λ*}^*ζ*^ is a sequence space over C*;
- if λ* < μ* < ω*, then {μ*}^*ζ*^ < {λ*}^*ζ*^;
- *I* is an index set, if *λ*_i_* are sequence spaces, and if λ* : = ⋃_*i*∈*I*_ *λ*_i_*, then 〈λ*〉^*ζ*^ = ⋂_*i*∈*I*_{*λ*_i_*}^*ζ*^, where the notation ^"^〈〉^"^ stands for the span of linear subspace over C*;
- λ* ⊂ {λ*}^*ζζ*^ : = ({λ*}^*ζ*^)^*ζ*^.

**Proof**

The case (b) obviously is true, and (a) follows from *l*_∞_* < cs* < bs*. We only show the cases (c) and (d) taking *ζ* = *alpha*. The rest of the parts can be obtained in a similar way.

(c) Now, as an immediate consequence *λ*_i_* ⊂ 〈λ*〉 the following 〈λ*〉^*α*^ ⊂ {*λ*_i_*}^*α*^ and 〈λ*〉^*α*^ ⊂ ⋂_*i*∈*I*_{*λ*_i_*}^*α*^ hold by (b). On the other hand, if *y* ∈ ⋂_*i*∈*I*_{*λ*_i_*}^*α*^, that is, *y* ∈ {*λ*_i_*}^*α*^, then *x*⊙*y* ∈ *l*_1_* for all *x* ∈ λ* and therefore *y* ∈ {λ*}^*α*^ ⊂ 〈λ*〉^*α*^.
(d) We can deduce λ* ⊂ {λ*}^*ζζ*^. Let *w* ∈ λ*; then *w*⊙*z* ∈ *l*_1_* for all *z* ∈ {λ*}^*α*^; thus *w* ∈ {λ*}^*ζζ*^ and λ* ⊂ {λ*}^*ζζ*^ by (a).

Here λ* ≠ {λ*}^*ζζ*^ as we get from Theorem 17 (a) in the case of *ζ* = *β* and λ* : = *c*_0_*. We have {*c*_0_*}^*ββ*^ = *l*_∞_* ≠ *c*_0_*. This remark gives rise to the following definition.

**Definition 19 (*ζ*-space, Köthe space).**

Let *ζ* ∈ {*α*, *β*, *γ*} and let λ* be a sequence space over the field C*. λ* is called *ζ*-space if λ* = {λ*}^*ζζ*^. Further, an *α*-space is also called a Köthe space or perfect sequence space.

From Remark 18 (d) and (b) we obtain immediately the following remark.

**Remark 20 .**

If λ* is a sequence space over the field C* and (*ζ* ∈ {*α*, *β*, *γ*}), then {λ*}^*ζ*^ is a *ζ*-space; that is, {λ*}^*ζ*^ = {λ*}^*ζζζ*^.

Now we look for sufficient conditions for {λ*}^*α*^ = {λ*}^*β*^ = {λ*}^*γ*^. This gives rise to the notion of solidity.

**Definition 21 (solid sequence space).**

Let *X* be a sequence space. Then *X* is called solid if

(40) $$\begin{array}{c} \left\{u=\left(u_{k}\right)\in \omega^{*}:\exists \left(x_{k}\right)\in X\;\;\forall k\in N:\ddot{||}u_{k}\ddot{||}\;\ddot{\leq }\;\ddot{||}x_{k}\ddot{||}\right\}\subset X. \end{array}$$

**Theorem 22 .**

Let λ* be a sequence space over the field C*. Then λ* is solid if and only if

(41) $$\begin{array}{c} \left\{y=\left(y_{k}\right)\in \omega^{*}:\exists \left(x_{k}\right)\in \lambda^{*}\;\;\forall k\in N:\ddot{||}y_{k}\ddot{||}\;=\;\ddot{||}x_{k}\ddot{||}\right\}\subset \lambda^{*}. \end{array}$$

**Proof**

Let *u* = (*u*_*k*_) ∈ ω* and *x* = (*x*_*k*_) ∈ λ* with $\ddot{||}u_{k}\ddot{||}\;\ddot{\leq }\;\ddot{||}x_{k}\ddot{||}$ be given. Further, let $(x_{k})=({\dot{\xi }}_{k},{\ddot{\eta }}_{k})\in \omega^{*}$, where *ξ*_*k*_, *η*_*k*_ ∈ *R*. Obviously the condition $\bar{x}=(\bar{x_{k}})=({\dot{\xi }}_{k},\ddot{-}\;{\ddot{\eta }}_{k})\in \lambda^{*}$ holds because $\ddot{||}x_{k}\ddot{||}=\ddot{||}\bar{x_{k}}\ddot{||}$. Let $u_{k}=({\dot{\psi }}_{k},{\ddot{\delta }}_{k})\in \omega^{*}$, where *ψ*_*k*_, *δ*_*k*_ ∈ *R*. We obtain $\beta \left\{\sqrt{\psi_{k}^{2}+\delta_{k}^{2}}\right\}\leq \beta \left\{\sqrt{\xi_{k}^{2}+\eta_{k}^{2}}\right\}$ so *ψ*_*k*_^2^ + *δ*_*k*_^2^ ≤ *ξ*_*k*_^2^ + *η*_*k*_^2^ since $\ddot{||}u_{k}\ddot{||}\;\ddot{\leq }\;\ddot{||}x_{k}\ddot{||}$ for all *k* ∈ *N*. We may choose a real number *γ*_*k*_ ∈ *R* with *ψ*_*k*_^2^ + *γ*_*k*_^2^ = *ξ*_*k*_^2^ + *η*_*k*_^2^ and consider *w* = (*w*_*k*_) defined by $\left(w_{k})=({\dot{\psi }}_{k},{\ddot{\gamma }}_{k}\right)$. Then $\ddot{||}w_{k}\ddot{||}\;=\;\ddot{||}x_{k}\ddot{||}$; thus *w* ∈ λ* and $\bar{w}=(\bar{w_{k}})\in \lambda^{*}$. From Definition 7 we get ${\dot{\psi }}_{k}=(w⊕\bar{w})\dot{/}\dot{2}\in \lambda^{*}$. Determining *ζ*_*k*_ ∈ *R* such that *ζ*_*k*_^2^ + *δ*_*k*_^2^ = *ξ*_*k*_^2^ + *η*_*k*_^2^ it follows similarly that $\mu =(\mu_{k})=({\dot{\zeta }}_{k},{\ddot{\delta }}_{k})\in \omega^{*}$ and $\bar{\mu }=(\bar{\mu_{k}})\in \lambda^{*}$, which imply ${\ddot{\delta }}_{k}=(\mu ⊖\bar{\mu })\ddot{/}\ddot{2}\in \lambda^{*}$. Hence $u_{k}=({\dot{\psi }}_{k},{\ddot{\delta }}_{k})\in \lambda^{*}$. This step completes the proof.

**Theorem 23 .**

Consider that λ* < ω* is any sequence space over the field C*; then the following statements hold.

- If λ* is a Köthe space, then λ* is solid.
- If λ* is solid, then {λ*}^*α*^ = {λ*}^*β*^ = {λ*}^*γ*^.
- If λ* is a Köthe space, then λ* is a *ζ*-space.

**Proof**

Let λ* < ω* be a sequence space over the field C*.

(a) If λ* is a Köthe space and *u* ∈ ω*, then *u* ∈ {λ*}^*αα*^ if and only if the condition *u*⊙*z* ∈ *l*_1_* holds for all *z* ∈ {λ*}^*α*^. Besides this we obtain $\ddot{||}v_{k}\ddot{||}=\ddot{||}u_{k}\ddot{||}$ for *u* = (*u*_*k*_) ∈ λ* and *v* = (*v*_*k*_) ∈ ω* and the statement
  (42) ∑∗ k||¨vk⊙zk||¨=∑∗ k||¨uk⊙zk||¨<∞
 holds for each *z* ∈ {λ*}^*α*^. Therefore *v*⊙*z* ∈ *l*_1_*. Hence *w* ∈ λ* and λ* is solid over the field C*.
(b) Consider that λ* is a solid sequence space over C*. To show {λ*}^*α*^ = {λ*}^*β*^ = {λ*}^*γ*^, it is sufficient condition to verify {λ*}^*γ*^ < {λ*}^*α*^ in Remark 18 (a). So, let *v* = (*v*_*k*_) ∈ {λ*}^*γ*^; that is,
  (43) sup⁡n∈N||¨∑∗ k=0     n(uk⊙vk)||¨<∞  for every u=(uk)∈λ∗.
 By taking into account solidness of λ*, for *z* = (*z*_*k*_) ∈ λ*, the condition $\ddot{||}z_{k}\ddot{||}\;\;=\ddot{||}u_{k}\ddot{||}$ holds and there exists a sequence *u* = (*u*_*k*_) ∈ λ* for all *k* ∈ *N*. Therefore by combining this with the inclusion (43) we deduce that the condition
  (44) ∑∗ k=0N||¨uk⊙vk||¨=∑∗ k=0 N||¨zk⊙vk||¨=||¨∑∗ k=0 Nzk⊙vk||¨≤¨ sup⁡n∈N ||¨∑∗ k=0 nzk⊙vk||¨<∞
 holds and *u*⊙*v* ∈ *l*_1_* < cs*. Hence *v* ∈ {λ*}^*α*^ and {λ*}^*γ*^ < {λ*}^*α*^.
(c) This is an obvious consequence of Remark 20 and the cases (a)-(b) in Theorem 23.

**Theorem 24 .**

The following statements hold.

- The sets φ*, ω*, *l*_p_*, *c*_0_*, and *l*_∞_* of sequences are solid.
- The sets c* and bv* of sequences are not solid; therefore none of them is a Köthe space.
- For each *ζ* ∈ {*α*, *β*, *γ*}, then
(i)
{*l*_1_*}^*ζ*^ = *l*_∞_* and {*l*_∞_*}^*ζ*^ = *l*_1_*;
(ii)
{ω*}^*ζ*^ = φ* and {φ*}^*ζ*^ = ω*.
- If *ζ* ∈ {*α*, *β*, *γ*} and *c*_0_* < μ* < *l*_∞_*, then {μ*}^*ζ*^ = *l*_1_* and μ* ⊂ {μ*}^*ζζ*^ = *l*_∞_*. In particular, {*c*_0_*}^*ζ*^ = {c*}^*ζ*^ = *l*_1_*, and each of c*, *c*_0_* is not a *ζ*-space.

**Proof**

That the specified spaces in cases (a-b) are solid is an immediate consequence of their definition. Additionally, the cases (i-ii) of (c) can be obtained Theorem 17 and Remark 16 (d). Since *c*_0_* and *l*_∞_* are solid, we know that {*c*_0_*}^*ζ*^ = {*l*_∞_*}^*ζ*^ = *l*_1_*. So the statements in (d) are obtained from Remark 18 (b).

Next, we determine the *ζ*-duals of the spaces cs*, bs*, bv*, and *bv*_0_*. We will find that none of these sequence spaces is solid; in particular, none of them is a Köthe space.

**Theorem 25 .**

The following statements hold:

- {cs*}^*α*^ = {bv*}^*α*^ = {*bv*_0_*}^*α*^ = *l*_1_*; {*bv*_0_*} = bv*∩*c*_0_*,
- {cs*}^*β*^ = bv*, {bv*}^*β*^ = cs*, {*bv*_0_*}^*β*^ = bs*, {bs*}^*β*^ = *bv*_0_*;
- {cs*}^*γ*^ = bv*, {bv*}^*γ*^ = bs*, {*bv*_0_*}^*γ*^ = bs*, {bs*}^*γ*^ = bv*.

In particular, the sets cs*, bs*, bv*, and *bv*_0_* of sequences are *β*-spaces (*ζ* = beta), but they are not Köthe spaces. Moreover, the sets bs* and bv* of sequences are *γ*-spaces (*ζ* = gamma), whereas both cs* and *bv*_0_* are not *γ*-spaces. None of the spaces cs*, bs*, bv*, and *bv*_0_* is solid.

**Proof**

We prove the cases for the spaces {cs*}^*ζ*^, *ζ* ∈ {*α*, *β*, *γ*}, and the proofs of all other cases are quite similar.

(a) Let *x* = (*x*_*k*_) ∈ cs* and *y* = (*y*_*k*_) ∈ *l*_1_*. Then,

(45) ∑∗ k||¨yk⊙xk||¨ ≤¨ ||x||cs∗ ×¨∑∗ k||¨yk||¨ < ∞ , ∀k∈N,

where ||*x*||_cs_* is defined by (27). Therefore, *y* ∈ {cs*}^*α*^ which gives the fact that *l*_1_* ⊂ {cs*}^*α*^.

Conversely, suppose that *y* = (*y*_*k*_) ∈ {cs*}^*α*^∖*l*_1_*. Then to every natural number *p* we can construct an index sequence (*n*_*p*_) with *n*_*p*_ < *n*_*p*+1_ and ∑∗∑k=np+1np+1||¨yk||¨ <¨ 4¨p¨ for all *p* ∈ *N*. Define *x* = (*x*_*k*_) by

(46) $$\begin{array}{c} x_{k}≔\{\begin{array}{cc} {\left(\ddot{-}\ddot{1}\right)}^{\ddot{k}}\ddot{/}{\ddot{2}}^{\ddot{p}}, & n_{p}<k\leq n_{p+1},\\ \ddot{0}, & \text{others}. \end{array} \end{array}$$

By using the algebraic operations in (4) and Remark 3 the *β*-division ${\left(\ddot{-}\ddot{1}\right)}^{\ddot{k}}\ddot{/}{\ddot{2}}^{\ddot{p}}$ can be evaluated as *β*{(−1)^*k*^2^−*p*^} for *n*_*p*_ < *k* ≤ *n*_*p*+1_. Then *x* = (*x*_*k*_) ∈ cs*. According to the choice of *n*_*p*_, the inequalities

(47) ∑∗ k||¨yk⊙xk||¨ ≥¨ ∑∗ p2¨−¨p¨ ×¨∑∗ k=np+1np+1||¨yk||¨ ≥¨  ∑∗ p2¨p¨=∞

hold, where the sum ∑∗∑p2¨p¨=β{∑pβ-1(2¨p¨)}=β{∑p2p}  ∗-diverges since the classical geometric series ∑_*p*_2^*p*^ diverges. Thus, *x*⊙*y* ∉ *l*_1_*, which implies *y* ∉ {cs*}^*α*^. This contradicts the fact that *y* ∈ {cs*}^*α*^. Therefore {cs*}^*α*^ ⊂ *l*_1_*.

The condition {*bv*_0_*}^*α*^ ⊂ *l*_1_* holds as well if we take the sequence (*x*_*k*_) by

(48) $$\begin{array}{c} x_{k}≔\{\begin{array}{cc} {\left(\ddot{2}\right)}^{\ddot{-}\ddot{p}}, & n_{p}<k\leq n_{p+1},\\ \ddot{0}, & \text{others}. \end{array} \end{array}$$

(b) Let *u* = (*u*_*k*_) ∈ {cs*}^*β*^ and *w* = (*w*_*k*_) ∈ *c*_0_*. Define the sequence *v* = (*v*_*k*_) ∈ cs* by *v*_*k*_ = (*w*_*k*_ ⊖ *w*_*k*+1_) for all *k* ∈ *N*. Therefore, _∗_∑∑_*k*_⊙  *v*_*k*_  ∗-converges, but

(49) ∑∗ k=0 n  (wk⊖wk+1)⊙uk  =[∑∗ k=0 n−1wk⊙(uk⊖uk−1)]⊖wn+1⊙un

and the inclusion *l*_1_* ⊂ cs* yields (*u*_*k*_) ∈ {cs*}^*β*^ ⊂ {*l*_1_*}^*β*^ = *l*_∞_*. Then we derive by passing to the ∗-limit in (49) as *n* → *∞* which implies that

(50) ∑∗ k=0 ∞(wk⊖wk+1)⊙uk=∑∗ k=0 ∞wk⊙(uk⊖uk−1)

for every *k* ∈ *N*. Hence, (*u*_*k*_ ⊖ *u*_*k*−1_) ∈ {*c*_0_*}^*β*^ = {*c*_0_*}^*α*^ = *l*_1_*; that is, *u* ∈ bv*. Therefore, {cs*}^*β*^⊆bv*.

Conversely, suppose that *u* = (*u*_*k*_) ∈ bv*. Then, (*u*_*k*_ ⊖ *u*_*k*−1_) ∈ *l*_1_*. Further, if *v* = (*v*_*k*_) ∈ cs*, the sequence (*w*_*n*_) defined by *w*_*n*_ = _∗_∑∑_*k*=0_^*n*^ *v*_*k*_ for all *k* ∈ *N* is an element of the space c*. Since {c*}^*α*^ = *l*_1_*, the *N*-series _∗_∑∑_*k*_ *w*_*k*_⊙(*u*_*k*_ ⊖ *u*_*k*−1_) is ∗-convergent. Also, we have

(51) ∑∗ k=m n(wk⊖wk+1)⊙uk ≤¨ [∑∗ k=m n−1wk⊙(uk⊖uk−1)]⊕(wn⊙un)⊖(wm−1⊙um).

Since (*w*_*n*_) ∈ c* and (*u*_*k*_) ∈ bv* ⊂ c*, the right-hand side of inequality (51) ∗-converges to $\ddot{0}$ as *m*, *n* → *∞*. Hence, the series _∗_∑∑_*k*=0_^*∞*^(*w*_*k*_ ⊖ *w*_*k*+1_)⊙*u*_*k*_ or _∗_∑∑_*k*=0_^*∞*^ *u*_*k*_⊙*v*_*k*_  ∗-converges. Hence bv*⊆{cs*}^*β*^.

(c) By using (a), it is known that bv*⊆{cs*}^*β*^ and since {cs*}^*β*^ ⊂ {cs*}^*γ*^, bv* ⊂ {cs*}^*γ*^. We need to show that {cs*}^*γ*^ ⊂ bv*. Let *u* = (*u*_*n*_) ∈ {cs*}^*γ*^ and *v* = (*v*_*n*_) ∈ *c*_0_*. Then, for the sequence (*w*_*n*_) ∈ cs* defined by *w*_*n*_ = (*v*_*n*_ ⊖ *v*_*n*+1_) for all *n* ∈ *N*, we can find a non-Newtonian real number $\ddot{K}\;\ddot{>}\;\ddot{0}$ such that ||¨∑∗∑k=0nuk⊙wk||¨ ≤¨ K¨ for all *n* ∈ *N*. Since (*v*_*n*_) ∈ *c*_0_* and (*u*_*n*_)∈{cs*}^*γ*^ ⊂ *l*_∞_*, there exists a non-Newtonian real number $\ddot{M}\;\ddot{>}\;\ddot{0}$ such that $\ddot{||}u_{n}⊙v_{n}\ddot{||}\;\ddot{\leq }\;\ddot{M}$ for all *n* ∈ *N*. Therefore,

(52) ||¨∑∗ k=0 n(uk⊖uk−1)⊙vk||¨ ≤¨ ||¨∑∗ k=0 n+1uk⊙(vk⊖vk+1)||¨+¨ ||¨vn+2⊙un+1||¨ ≤¨ K¨+¨M¨.

Hence (*u*_*k*_ ⊖ *u*_*k*−1_) ∈ {*c*_0_*}^*γ*^ = {*c*_0_*}^*α*^ = *l*_1_*; that is, (*u*_*n*_) ∈ bv*. Therefore, since the inclusion {cs*}^*γ*^ ⊂ bv* holds, we conclude that {cs*}^*γ*^ = bv*, as desired.

## 5. Concluding Remarks

Although all arithmetics are isomorphic, only by distinguishing among them, we do obtain suitable tools for constructing all the non-Newtonian calculi. But the usefulness of arithmetics is not limited to the construction of calculi; we believe there is a more fundamental reason for considering alternative arithmetics; they may also be helpful in developing and understanding new systems of measurement that could yield simpler physical laws.

We trust that the basic ideas of the non-Newtonian calculus have been presented in sufficient detail to enable interested persons to develop the theory in various directions. For example, consider the concept the* average speed*. The definition “distance traveled per unit time” is incomplete because it fails to provide a method of determining the average speed of an accelerated particle. The definition “distance divided by time,” though not incorrect, is a gross oversimplification that fails to reveal the underlying issues. Fortunately there is a completely satisfactory definition, which undoubtedly was known to Galileo. Then we isolate a constant in each given uniform motion by defining speed to be the distance traveled in any unit time-interval. Finally, for a particle that moves nonuniformly in a distance *d* in time *t*, we define the average speed to be the speed that a particle in uniform motion must have in order to travel a distance *d* in time *t*. In our opinion, neither the simplicity nor the obviousness of the answer, *d*/*t*, justifies its use as the definition of average speed (cf. [12]).

Of course, we can only speculate as to future applications of the non-Newtonian calculi. Perhaps they can be used to define new scientific concepts, to yield new or simpler scientific laws, to solve heretofore unsolved problems, or to formulate and solve new problems. For example, one constructs non-Newtonian calculi of functions of two or more real variables by choosing an arithmetic for each axis. It might even be profitable to seek deeper connections among the corresponding operators of the calculi. Like as, some of them are postponed to our future works.
